# Dr. Harvey Scott

**Published:** 1896-03

**Authors:** 


### Obituary.

DIED, September 23, 1895, in Toledo, Ohio, Dr. Harvey Scott, of Lancaster, Ohio.

Dr. Scott was born at Old Town, Green Co., Ohio, January 30, 1809. Until his seventeenth year he was with his father who was a farmer, and endured the hardships, privation, and struggle, of the frontier life. His early education was received in a log school-house of the pioneer age. In 1826 he left his father's house and made his home with a family in South Charleston, Clark Co., Ohio, where he had further educational advantages, selecting a light manufacturer's trade. In 1833 he began the study of the medical profession, and in 1836 began its practice. Two years later he changed his profession to dentistry and in April, 1839, located in Lancaster, Ohio, where he resided until 1891, when he went to Toledo, Ohio, to live with his daughter, Mrs. Charles Hutchinson.

In November, 1830, he was married to Lydia Ann Melton, who died June, 1841. In April he was again married to Priscilla Ann Crook, of Lancaster, who was the mother of his children; her death occurred July, 1873. In 1875 he married his third wife. His family consisted of six children. Dr. Scott, especially in middle life, was quite inclined to literary pursuits. For ten years, from 1859, he was the editor of a paper in Lancaster.

In 1876 he wrote the history of Fairfield Co., which was regarded up to the present as containing the most complete and accurate data in regard to county affairs that is obtainable. He was a close student of human nature; was affable and a friend of all, and he enjoyed the esteem and confidence of all his acquaintances.

Considering his advanced age, 86 years, he enjoyed very good health. About eight months before his death a breaking down of the general system made its appearance, and though he had wonderful vitality, the malady made slow progress, until life peacefully passed away and an honorable life was ended.

Dr. Scott was one of the pioneer dentists of Ohio, and well did he maintain the status and dignity of the profession, and though he did not add very largely to its literature, owing to his being occupied in other directions, he never did aught that reflected anything but honor upon his chosen profession. His life was one well worthy of emulation.

